# Novel PAradigm to improve Inflammatory burden in end stage Renal disease (rePAIR): study protocol for a randomized controlled trial

**DOI:** 10.1186/s13063-018-2760-y

**Published:** 2018-07-11

**Authors:** Ruchir Trivedi, George Fares, Victoria Barany Nunez, Ryan Campbell, Megyn Clement, Joseph Burleson, Jonathan Himmelfarb, Effie Ioannidou

**Affiliations:** 1 Division of Nephrology, UCONN Health, Farmington, CT USA; 2 Bay State Medical Center, Springfield, MA USA; 3 Advanced Education in General Dentistry, UCONN Heath, Farmington, CT USA; 4 Clinical Research Center, UCONN Health, Farmington, CT USA; 5 Community Medicine and Health Care, School of Medicine, UCONN Heath, Farmington, CT USA; 6 Kidney Research Institute, School of Medicine, University of Washington, Seattle, WA USA; 7 Division of Periodontology, School of Dental Medicine, UCONN Health, Farmington, CT USA

## Abstract

**Background:**

Given the importance of inflammation as a predictor of poor outcomes in End Stage Renal Disease (ESRD), reductions in inflammatory biomarkers have been proposed as a critical target in this population. This study targets chronic periodontitis, an oral inflammatory disease of microbial etiology causing persistent inflammation in ESRD. Unlike the previously reported episodic periodontal interventions, we propose to control periodontal inflammation with a continuous maintenance and oral health behavior modifications. We hypothesize that this strategy will improve systemic inflammation and oxidative stress, oral health and quality of life within the 6-month observation period.

**Methods:**

The rePAIR (novel **PA**radigm to improve **I**nflammatory burden in ES**R**D) study is a pilot and feasibility, parallel-arm, and randomized controlled clinical trial that will recruit 72 ESRD subjects with periodontitis in a model of computerized block randomization. This trial aims to compare the effect of standard-of-care vs. repeated non-surgical periodontal therapy on systemic and oral inflammatory burden. This trial will recruit ESRD adult patients with periodontitis older than 21 years old with a minimum of 12 teeth and no history of periodontal treatment within a year. The trial will examine serum C-reactive protein (CRP) (primary outcome) as a biomarker of inflammation as well as interleukin-6 (IL-6), F2 isofurans and F2 isoprostanes (secondary outcomes) and compare their difference between groups from baseline to 6 months. The trial will also compare the difference between groups in patient-centered and clinical oral outcomes from baseline to 6 months.

**Discussion:**

The trial follows a rigorous and transparent study design capturing elements such as pre-specified eligibility criteria, pre-specified primary and secondary outcomes, detailed intervention description to allow replication, intervention random allocation and concealment, blinding in outcome assessment, appropriate sample size calculations, explanation of interim analysis, as per CONSORT Guidelines. Further, gender diversity is secured not only at recruitment but also throughout the trial and during the analysis. Therefore, treatment response outcomes will be examined per gender category. In order to manage anticipated problems, the protocol has included alternative approaches.

**Trial registration:**

ClinicalTrials.gov, NCT03241511. Registered on 7 August 2017.

**Electronic supplementary material:**

The online version of this article (10.1186/s13063-018-2760-y) contains supplementary material, which is available to authorized users.

## Background

Within the last decades, the high inflammatory burden in ESRD has been attributed to the “uremic puzzle” in which pieces developing and connecting in an intricate manner [1] contribute to cardiovascular disease (CVD) mortality. We now know that the complexity of the “uremic puzzle” extends past the Framingham CVD risk factors paradigms and involves systemic inflammation and oxidative stress as variables strongly associated with poor CVD outcomes in Chronic Kidney Disease (CKD) [1]. Given the importance of inflammation as a predictor of cardiovascular mortality in ESRD [2, 3], reductions in biochemical inflammatory markers have been proposed as critical target outcomes in this population [4]. Several anti-inflammatory strategies have been utilized in this direction assessing nutritional [5] as well as pharmacological interventions [6–8]. Although the results of these trials hold promise, many investigators recognized different sources of inflammation in these patients, which need to be resolved in order to achieve the most optimal responses [3, 9]. As expected, the inflammatory modulation requires concurrent therapy of the multiple comorbidities, which characterize this population [10–12].

This study focuses on targeting chronic periodontitis, an oral inflammatory disease of microbial etiology, which causes connective tissue and bone destruction and, consequently, leads to tooth loss [13] . Chronic periodontitis is associated with increased risk for atherosclerosis, adverse pregnancy outcomes, rheumatoid arthritis, chronic obstructive pulmonary disease and aspiration pneumonia [14–17]. Although recent epidemiological evidence showed a severe periodontitis prevalence of ~ 8.5% in the general US population [18], this prevalence increased up to ~ 40% in some racial groups with CKD and exceeds 50% in ESRD [19].

Given that periodontitis is recognized as a cause of persistent inflammation in ESRD [20] and is associated with increased CVD mortality risk [21], untreated periodontitis should be considered a forgotten comorbidity in ESRD.

Evidence deriving from a long-term randomized controlled trial on standard-of-care periodontal treatment in ESRD revealed no significant effect on inflammatory changes [22] possibly due to the episodic approach and the lack of repeated maintenance within the 6-month period, which failed to achieve clinically acceptable periodontal endpoints. Although results from this trial hold promise, modified and tailored periodontal therapy modalities might be more appropriate to control inflammatory burden in ESRD. Therefore, this tailored approach will tackle the forgotten comorbidity of periodontitis with emphasis on oral and systemic outcomes.

Well-designed interventional studies are essential, therefore, for the development of systematic periodontal treatment and maintenance protocols in ESRD. Since kidney transplant is considered the highly preferred modality of renal replacement, disease-free oral environment is a major priority, relevant to the approximately 19% of dialysis patients receiving a kidney transplant within the first year of dialysis [23].

## Research hypothesis and specific aims

We hypothesize that there is a difference in the response to repeated vs. standard-of-care periodontal interventions as measured by: a) patient-centered outcomes, b) inflammatory, oxidative stress and nutritional biomarkers as well as c) clinical oral outcomes. Given that positive oral response is required for systemic anti-inflammatory effects to occur, we will assess periodontal treatment effectiveness at oral level (Aim 1) and its correlation with systemic effects (Aim 2). This hypothesis will be tested by the following specific aims:

### Specific aim 1

We aim to compare the difference (Δ) in patient-centered as well as clinical oral outcomes between ESRD subjects receiving repeated (Test group) and the standard-of-care (Control) periodontal therapy. For this aim, we will measure and compare patient-centered outcome (Oral Health Impact Profile-14 survey) reflecting patient’s perspective on oral health at baseline and 6-months. The clinical oral outcomes will evaluate the clinical response and effectiveness of the intervention at each time point.

### Specific aim 2

We aim to compare the difference (Δ) in outcomes of systemic inflammation (C-reactive protein, Interleukin 6), oxidative stress (F2-isoprostanes, and isofurans) in the repeated vs. standard-of-care periodontal therapy in ESRD at baseline and 6-months. To accomplish this, we will collect blood samples from all patients at each time point. Serial changes of the inflammatory and oxidative stress markers will also be evaluated during the observational period and correlated with clinical disease markers.

## Methods

### Administrative information

The rePAIR (novel **PA**radigm to improve **I**nflammatory burden in ES**R**D) study is a pilot and feasibility, parallel-arm, and randomized controlled clinical trial that will recruit 72 ESRD subjects with periodontitis in a model of computerized block randomization. The trial is approved by UCONN Health IRB (IRB #16–111-1) and the DCI Administrative Review Office (ARO) (#2016–38) and registered at clinicaltrials.gov (NCT03241511) on August 7th, 2017. The trial is funded by NIH/NIDDK (Award #R21DK108076). Patient recruitment was initiated on November 7th, 2017 with expected enrollment completion in April 2019. The rePAIR trial protocol adhered to the SPIRIT 2013 statement and checklist as shown in Additional file 1.

### Study setting

The study is conducted at UCONN Health, Farmington, CT in collaboration with DCI Units in Farmington and Manchester, CT.

#### Population

This trial will recruit ESRD adult patients with periodontitis fulfilling the eligibility criteria below.

#### Inclusion criteria

Individuals will be included in the study if they are older than 21 years old; if they are ESRD on dialysis; if they have a minimum of 12 teeth; if they have chronic periodontitis as defined by the presence of at least two sites with CAL ≥ 4 mm or at least two sites with PD ≥ 5 mm not on the same tooth [24]; if they have no history of periodontal treatment within a year; if life expectancy is more than one year; or if they can provide consent form.

#### Exclusion criteria

Individuals will be excluded from the study if they had periodontal treatment within a year prior to the study initiation; if they have HIV/AIDS, or active malignancy; if they poorly adhere to dialysis treatment; in anticipation for kidney transplant during study period; if they are pregnant; if they have dementia; if they take anti-inflammatory medication, except aspirin≤325 mg/d; or if they use temporary catheter for dialysis access.

#### Intervention

The Test arm will receive modified periodontal treatment and maintenance sessions. Briefly, treatment sessions will include oral hygiene behavioral modification and scaling/root planing (removal of bacterial biofilm and calculus) in order to eliminate etiologic factors and control periodontal inflammation. More specifically, the behavioral interventions will include demonstration of oral hygiene instructions such as tooth brushing and flossing techniques. In order to standardize the oral hygiene session (intervention fidelity), the providers will be trained to follow specific oral hygiene guidelines with re-calibration on the protocol procedures every three months to maintain study quality control. Once the treatment sessions are completed, the subjects will be followed for six months and receive systematic supportive periodontal treatment (tooth cleanings with re-enforcement of oral hygiene) every other month and oral hygiene behavioral modification every month for six months. Outcomes will be assessed at baseline and the end of the trial as well as at 2- and 4-months.

#### Control

**Fig. 1:**
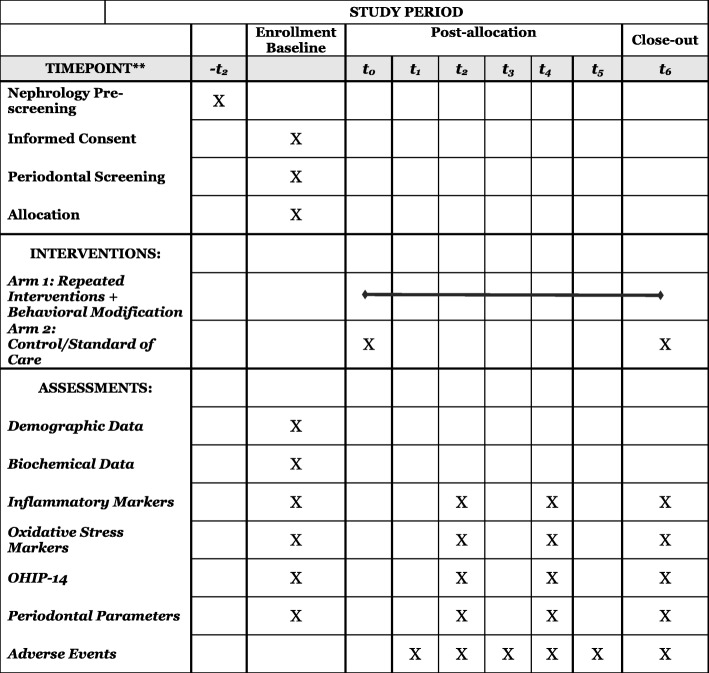
Standard protocol time points and items based on the SPIRIT guidelines. *Recommended content can be displayed using various schematic formats. See SPIRIT 2013 Explanation and Elaboration for examples from protocols. **List specific timepoints in this row

The Control arm will receive the standard-of-care therapy including scaling/root planing (removal of bacterial biofilm and calculus) plus a single session of oral hygiene modification followed by no additional maintenance sessions within the 6-month period (as shown in Fig. 1). Outcomes will be assessed at 2-, 4-, and 6-months.

#### Additional dental needs

Throughout the course of the study, additional dental needs of both arms (Test and Control) will be addressed with immediate referral to the Advanced Education in General Dentistry Residency Program at the UCONN Health or the subject’s own general dentist. In cases where the included teeth are restoratively compromised, referrals will include treatment decisions on primary or secondary caries and/or tooth fractures on any teeth.

#### Strategies to improve adherence

We systematically review our recruitment and retention rates as well as feedback from patients in the team’s bi-weekly meetings. For treatment fidelity, the study staff will be trained to follow an oral hygiene script and guidelines followed by re-calibration on the protocol procedures every three months to maintain study quality control. The OHIP-14 will be filled during oral hygiene session in an interactive interview form following provider training on the questionnaire administration to control for treatment fidelity.

#### Outcomes and rationale

1) Inflammatory biomarker (primary outcomes) and oxidative stress markers (secondary outcomes): We aim to measure serum CRP (primary outcome) as a biomarker of inflammation as well as IL-6, F2 isofurans and F2 isoprostanes (secondary outcomes) as markers of inflammation and oxidative stress linked to atherosclerosis and poor CVD outcomes in ESRD [25]. A single serum CRP elevated measure has been shown to predict poor outcomes and sudden death in ESRD [26]. Additional studies showed that IL-6 has been determined as a direct promoter of atherosclerosis and protein energy wasting through mechanisms of vascular calcification, muscle catabolism and cell aging [12] predicting poor outcomes in ESRD [26]. Moreover, we will also measure and explore F2 isoprostanes and isofurans, which are chemically stable, precise and easily detectable markers of lipid peroxidation [27] associated with poor outcomes in ESRD [1] but were never before measured after periodontal interventions in ESRD.

2) Patient-centered outcome (secondary outcome): The validated OHIP-14 aims to evaluate patient’s perception of oral status change [28] and predictably assess periodontal treatment effect on oral health quality of life [29, 30]. Oral health perception has been determined to be fair to poor in groups of ESRD patients [31]. The oral health related quality of life will be assessed with the OHIP-14 questionnaire [32]. The response codes for the items in this tool are based on a five-point scale, ranging from “never” to “all the time”.

3) Clinical periodontal parameters (secondary outcomes): Full mouth periodontal examination includes missing teeth, probing depth (PD), bleeding on probing (BOP), clinical attachment loss (CAL), and plaque score (PS) at six sites on all teeth. The above periodontal parameters are measured at baseline, 2-, 4- and 6-months. Changes in probing depth (ΔPD) and BOP (ΔBOP) between baseline and 6-months are evaluated to assess periodontal oral clinical response to periodontal intervention.

4) Adverse event frequency (secondary outcome): For this outcome, pain, swelling, number of analgesics used (continuous) and presence of oral ulcers (dichotomous) will be recorded. More specifically, pain and swelling will be measured by ordinal scale (10 cm horizontal Visual Analogue Scale, VAS).

### Study visits

Figure 1 presents the visit timeline per study arm.

### Power analysis

For power analysis, we assumed a Type I error rate α = 0.05 and a Type II error, β =0.20 (1-β = 0.80) [33]. Based on the preliminary data analysis of independent CRP changes, and after adjusting for time effect, we accepted a medium effect size (d = 0.67). Hence, within the pilot grant limitations, we calculated a sample size of 28 per arm. Given a reported 30% attrition rate in ESRD research, we aim to enroll up to 36 per arm. More importantly, pilot data from this trial will enable calculation of appropriate effect estimate and power analysis of a future large trial.

### Recruitment and consent procedures

**Fig. 2:**
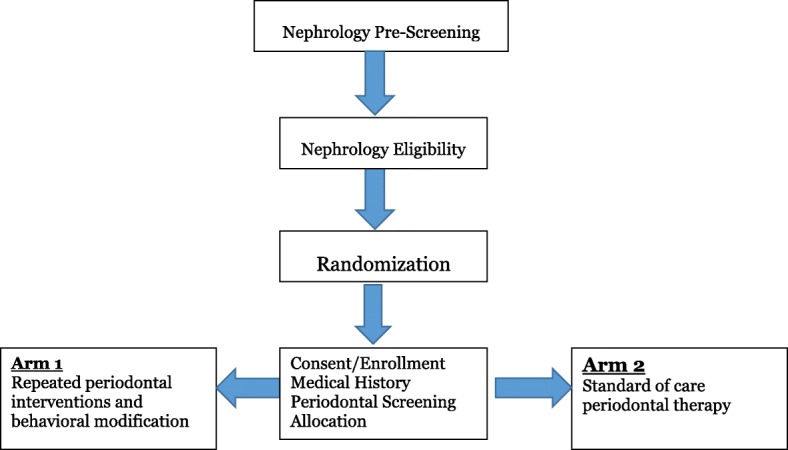
Study Flow Chart

Patient recruitment was initiated on November 7th, 2017 with expected enrollment completion in April 2019. The trial is currently recruiting patients at UCONN Dialysis/DCI and DCI Manchester. The recruitment strategy includes study fliers posted in different locations including UCONN Health Nephrology, Dental Clinics and Dialysis Outpatient Units. All recruitment strategies and material were approved by UCONN Institutional Review Board (IRB) and DCI ARO. Figure 2 represents study flow chart.

The initial patient selection is based upon administrative and medical record review by the nurse manager with the utilization of the study’s pre-screening form. The nephrologist reviews the patient selection and, based on clinical judgment and the pre-screening criteria makes the recommendation. We have also developed recruitment tools (pre-screening forms, study brochures), which are practical and easy to implement in order to assist the nephrologist and dialysis personnel during the pre-screening process. Once the pre-screening process is completed and the patient agrees to be approached, the study coordinator meets with the ESRD patient to present the study, the overall risk/benefits analysis, written consent is sought from each of the participants. The consent process may be extended to more than one meeting given the patient’s level of fatigue [34].

### Randomization and allocation

The study follows a block randomization scheme. Blocks will be determined as per 2X2X2 *a priori* categorization of age, race and gender. More specifically, the participants in two dialysis units (UCONN Dialysis/DCI and DCI Manchester) will be randomly assigned to the study arms using a computer randomization program supervised by the study’s biostatistician. Once sequences are determined, they remained concealed in opaque envelopes and opened prior to enrollment in the unit by the study coordinator.

### Examiner calibration

The calibrated dental hygienist practices under the supervision of the Principle Investigator (PI). Following the calibration process, there was an agreement within ±1 mm of 96%.

### Blinding

At 6-month visit, the final assessment will be conducted by the PI, who will be blinded to the study arm. Unblinding will be permissible in case of serious adverse events. The PI will be informed of the arm allocation to be able to appropriately respond to the management of the adverse event.108.

Data and sample collection.

#### Medical data

Demographic, anthropometric, medical history data will be collected using a standardized data extraction form at baseline. The biochemical data will include serum albumin, dialysis adequacy (Kt/V), vitamin D levels. Additional demographic and medical data such as race, history and duration of diabetes, diabetic control, smoking history, history of CVD, body mass index (BMI), history of peripheral arterial disease and comorbid conditions will be collected. In order to assess the magnitude of comorbidities, we will use the Charlson Comorbidity Score, which has been validated in ESRD and found appropriate to assess comorbidity prognostic impact [35]. Biochemical data will be examined at every study time point to assess medical changes.

#### Periodontal data

Full mouth periodontal examination will include: numbers of missing teeth, pocket depth (PD), clinical attachment level (CAL), plaque score (PS), bleeding on probing (BOP). PS (O’Leary) is a dichotomous plaque measure with the use of disclosing solution at six sites of all teeth. Pocket depth (PD) is the distance from the gingival margin to the base of the pocket is measured in mm. Bleeding on Probing (BOP) is scored after probing depth measurements are taken. Clinical attachment level (CAL) represents the distance from the cemento-enamel junction to the base of the pocket in mm. For all measures, six sites around each tooth will be examined: mesial-buccal, buccal, distal-buccal, distal-lingual, lingual and mesial-lingual.

#### Blood collection and analyses

Blood samples will be drawn prior to dialysis session at each participating dialysis unit. Samples will be centrifuged at 3000 rpm for 15 min and then transported on ice. Samples will be coded and stored at − 70 °C. IL-6 and hsCRP levels will analyzed in duplicate by ELISA with kits from BioSource International (Carmillo, CA) and Diagnostic Systems Laboratories (Webster, TX). Oxidative stress markers will be quantified by simultaneous measurements of F2-isoprostane and isofuran concentrations with gas chromatography as described before [6].

### Statistical methods

All analyses will follow the intent-to-treat principle. Given an attrition of rate of approximately 30% in the dialysis population, missing data will likely be from different sources (e. g., failure to complete questionnaire, study dropout, death). We will, therefore, utilize the multiple imputation algorithms of the IBM SPSS Missing Values 22 software [36, 37] to impute missing data. Data will be screened as recommended [38]. Standard diagnostic procedures will examine whether multivariate outliers exist, as well test for deviations from normality and linearity among dependent measures. When continuous measures are skewed, linear transformation will be attempted in order to preserve the inherent power of the continuous metric [39]. Descriptive analysis for all four time points will be including continuous measures with means, medians and standard deviations. Given OHIP-14 responses are ordinal, we will calculate the responses based on the “simple count”, score frequencies as used before [40] and analyzed with non-parametric methods. Spearman correlation analysis will be used to test associations between clinical parameters and patient’s oral health perception. In order to assess the effect of the confounding factors on oral response, univariate model will correlate PD and CAL changes with the baseline parameters, demographic, socioeconomic status variables, as well as diabetic control, dialysis adequacy and vintage, vitamin D levels.

Exploratory univariate analysis and test statistics will be conducted to examine variable distribution. Outliers defined as more than two standard deviations from the mean will be detected. All biomarker variables will be tested for normality and logarithmically transformed if not normal. All analyses will include means and standard deviations for continuous variables and percentages for categorical variables. For skewed variables, analyses will show median and interquartile ranges. CRP and IL-6 changes (ΔCRP and ΔIL6) will be the dependent variables for hypothesis testing between the two arms. We will use a 3-level (time, subject, and cluster) hierarchical linear modeling technique [41] in the analysis of the data. The intercept (as a more robust indicator of initial baseline status) and slope (as an indicator for change, modeled as linear) are created for each subject. Predictors of inflammatory response will be assessed at subject level as well as subject nested within arm/recruitment site level. Additional secondary analyses will assess changes in the nutritional markers. The HLM will isolate predictors of inflammatory and oxidative stress response based on clinical phenotype, and medical confounders.

### Retention efforts

Physical limitations and fatigue, which may prevent this population from independent everyday transportation, have been identified as major reasons compromising research study participation [42]. Hence, to overcome these difficulties, we have developed a community-based model, which has been effective with the retention and adherence rates. Based on this model, we will utilize a mobile dental unit that facilitates examinations and periodontal therapy in the isolation room of each hemodialysis unit. The isolation room secures privacy for both patient and provider. We systematically review our recruitment and retention rates as well as feedback from patients in the team’s monthly meetings.

### Data and safety monitoring plan

The study data will be reviewed annually. Given that the clinical protocol procedures in the proposed study are standard of care procedures with minimal/slight increase over minimal risk, the individuals in the Data and Safety Monitoring Board (DSMB) will be the PI, the study coordinator, an institutional colleague, who is not involved in the study, and the IRB.

The data used for safety monitoring will be data related to serious adverse events, dropout rates and reasons for the dropouts, enrollment numbers, analysis of outcome data and its relationship to potential changes in study design, protocol deviations.

### Protection of confidentiality

The study will be conducted under the supervision of the PI, the co-Investigators and the collaborators. Best medical practices will followed during all procedures. All dialysis units are staffed with qualified and licensed personnel prepared to address emergencies. Further, nephrologist/attending is present at the dialysis units to clear the patient’s medical status. During the course of the study, a copy/summary of the monitor reports and/or minutes will be submitted to the IRB, Clinical Research Center (CRC) and NIH. The PI will evaluate the adverse events and determine whether the adverse events affect the risk/benefit ratio of the study and if modifications in the protocol and/or consent form are needed. In case of adverse events, the PI will report them to IRB, CRC, and NIH. Study data will be maintained in a separate research record. All materials and data will be coded with number combinations. The code linking the study data to identifiers will be in the PI’s computer protected by password. All study records will be coded in a locked cabinet in a secure area accessible only to research staff. A copy of the consent will be placed in the dialysis unit medical record. All electronic records (e.g. database, spreadsheets) will be password protected. Any computer hosting such files will have password protection to prevent access by unauthorized users. Access to study data will be restricted to the PI and Co-Is as well as the study key personnel. Data that will be shared with others will be coded to confidentiality protection.

### Study management

At the study initiation meeting, a delegation of responsibilities was carried out according to which, each investigator, and study personnel agreed to their assigned roles. The study PI has been in charge of the execution of this plan. Roles were assigned as justified in the research grant proposal. At the end of the initiation meeting, a study flow sheet was developed with all study procedures and practices. The flow sheet follows the study clinical schema as presented above. The flow sheet guarantees that all study activities are completed in the specific time points as described in the research strategy and the endpoints and outcomes are reached.

The study coordinator has been in charge of the regulatory management of the study, which includes the development of the regulatory binder with all essential documents. The regulatory binder includes study protocol, the study personnel human subject training log, the delegation of responsibilities form, the pre-screening and screening forms, consent forms, HIPAA forms, serious adverse events list, protocol deviation reports, and IRB approvals. The study subject chart contains all information about each study visit dated appropriately and precisely as well as a note declaring the procedures completed per visit. The pre-screening form is part of the chart. De-identified subjects that fail pre-screening are also maintained in a separate file. The study coordinator maintains a master subject log list, where all visits of all subjects are securely listed. This list guarantees accurate scheduling based on study time points.

The PI and the study coordinator are in charge for reporting the adverse events and also the follow up visit with the subject. Further, any protocol deviation is documented and reported. All laboratory assessments are maintained in a log list with specimen numbers and dates. An important part of the study is the quality management plan, which ensures study quality and adherence to the protocol standards. To achieve this, we have established an extensive monitoring plan to secure the study design, and achieve the endpoints and outcomes. Weekly study team communication and meetings are scheduled to prevent any misunderstandings or protocol gaps. The meetings help the team resolve problems and move to corrective actions at once before they become an established pattern. Every three months, the study subject charts are audited internally. To ensure the presence of consent form, pre-screen and screening form, lab requests, data collection forms, missed appointments and notes. Further, the electronic data files and all data variables are crosschecked with the source original data. The subject ID will be also crosschecked. Missing data will be left blank. Merging files of laboratory and clinical variables will be performed carefully based on ID variable.

## Study rigor and transparency

The trial follows a rigorous and transparent study design as requiring by the NIH capturing elements of trail design, pre-specified eligibility criteria, pre-specified primary and secondary outcomes, detailed intervention description to allow replication, intervention random allocation and concealment, blinding in outcome assessment, appropriate sample size calculations, explanation of interim analysis, as per CONSORT Guidelines [43, 44]. The proposed methods secures a robust and unbiased analysis as expected in a randomized controlled trial (RCT) and will ensure reproducibility of the experimental design [45]. With these efforts, we acknowledge the importance of research transparency through updated trial status, protocol, results and data reporting on Clinicaltrials.gov, as expected [46].

## Discussion

Despite periodontitis recognition as a critical public health problem (Healthy People 2020), periodontal disease awareness in ESRD populations is low. Therefore, the goal of this trial is to assess modifications in the standard of care periodontal practice as shaped by the needs of the medically compromised ESRD populations. The proposed study is innovative because it is the first study to:

- Assess the long-term effect of repeated oral health interventions on serial changes of systemic inflammatory and oxidative stress in ESRD patients.
- Offer a global testing of the hypothesis by focusing on patient-reported outcomes as supported by US Department of Health and Human Services, Food and Drug Administration guidelines on clinical studies (FDA guidelines, 2009) and the NIH roadmap for re-engineering the Clinical Research Enterprise (2002).
- Establish treatment protocol safety data by documenting adverse events within the observation period.
- Implement a systematic oral care model in an urban setting capturing ethnic and socioeconomic population diversity.
- In 6-month trial follow-up, we expect to find a significant anti-inflammatory effect as measured by changes in the serum CRP levels (primary outcome) as a result of repeated oral interventions compared to single, standard of care periodontal interventions. In addition, the direct anti-inflammatory contribution of the repeated periodontal intervention could be attributed to the clinical periodontal changes linking specific aims 1 and 2.

In order to manage anticipated problems, the protocol has included alternative approaches. More specifically, a possible failure to confirm the correlation between clinical periodontal and inflammatory markers may be attributed to the heterogeneity of inflammatory responses post-treatment as shown in the general population [47]. Although our analytical approach will include careful adjustment based on comorbidity score, we may still need to accept the presence of residual confounding in the analyses associated with disease severity. Given the complexity of the ESRD populations, additional testing will be performed on biomarker changes in correlation with periodontal disease severity. To prevent population selection bias based on socio-economic status or ethnic/racial characteristics in dialysis units, these variables will be included in the modeling for further adjustment. Protocol adherence and treatment fidelity will be monitored in order to minimize variations in the intervention implementation [48]. For this, we use treatment-standardized guidelines, quality assurance checks, frequent conferences and calibration meeting to discuss and control the intervention.

Patient’s perception of oral health status will be improved following treatment and continuous maintenance as shown before [29]. Non-surgical periodontal therapy in systemically healthy individuals controls periodontal inflammation, reduces PD and results in attachment gain [49]. Therefore we expect that Arm 1 will demonstrate a pronounced clinical response to periodontal treatment as an expected result of continuous care [50]. Some patients may still lose periodontal attachment during the observation period [51, 52]. In case of disease progression (increase of pocket depths more than 2 mm from baseline with BOP confirmed in two consecutive visits), the patients will be referred to the UCONN Health Periodontology clinic for additional care. Given the frequent vascular access infections, we anticipate use of systemic antibiotics during the course of the 6-month follow-up [53]. Hence, in this case, antibiotic frequency and dosage will be considered in the multivariate models.

This pioneer trial aims to directly address oral preventive practices leading to an infection free environment, promoting optimal oral hygiene, preventing recurrent periodontitis and controlling systemic inflammation [54, 55]. In our model, the dialysis outpatient centers could serve as a prototype for continuous in center oral health maintenance as shown below in our approach and recruitment strategy. If the feasibility of this model is established, then larger clinical trials may be developed to solidify the knowledge on effective approaches and guidelines targeting persistent inflammation in ESRD.

## Trial status

As of June 15th 2018, this trial is recruiting participants.

## Additional file

Additional file 1: SPIRIT 2013 Checklist: Recommended items to address in a clinical trial protocol and related documents*. (PDF 93 kb)
